# Glucose Ingestion Inhibits Endurance Exercise-Induced IL-6 Producing Macrophage Infiltration in Mice Muscle

**DOI:** 10.3390/nu11071496

**Published:** 2019-06-30

**Authors:** Takaki Tominaga, Sihui Ma, Kumiko Saitou, Katsuhiko Suzuki

**Affiliations:** 1 Graduate School of Sport Sciences, Waseda University, Tokorozawa 359-1192, Japan; 2 Neural Circuit Unit, Okinawa Institute of Science and Technology Graduate University (OIST), Okinawa 904-0495, Japan; 3 Faculty of Sport Sciences, Waseda University, Tokorozawa 359-1192, Japan

**Keywords:** endurance exercise, glucose ingestion, interleukin 6 (IL-6), macrophage, monocyte chemotactic protein (MCP)-1

## Abstract

Background: Carbohydrate (CHO) supplementation during exercise attenuates exercise-induced increases in plasma Interleukin (IL)-6 concentration. However, the effects of CHO supplementation on muscle IL-6 production during endurance exercise is controversial. The purpose of this study was to investigate the effects of CHO supplementation on muscle IL-6 production during endurance exercise with a special focus on the IL-6 producing cells. Methods: C57BL/6J mice were divided into three groups—sedentary with water ingestion group as the control (Con; *n* = 10), exercise with water ingestion group (Ex; *n* = 10), and exercise with 6% glucose ingestion group (Ex + glucose; *n* = 10). The Ex and Ex + glucose groups completed 3 h of treadmill running (24 m/min, 7% incline) and were sacrificed immediately after exercise. Results: The exercise-induced increases of plasma IL-6 concentration and gastrocnemius IL-6 gene expression were attenuated by glucose ingestion. However, the increases of soleus IL-6 gene expression and gastrocnemius and soleus IL-6 protein expression were not attenuated by glucose ingestion. Furthermore, we observed that macrophages that infiltrated muscle produce IL-6 and glucose ingestion attenuated the infiltration of IL-6-producing macrophages. Conclusion: This study revealed that infiltrating macrophages may be one type of IL-6-producing cells during endurance exercise, and the infiltration of these cells in muscle was attenuated by glucose ingestion. However, the effects of glucose ingestion on muscle IL-6 production were limited.

## 1. Introduction

Interleukin (IL)-6 is one of the cytokines which dramatically increases in blood circulation during endurance exercise [1,2,3]. IL-6 is representative of acute or chronic inflammatory status. IL-6 also induces anti-inflammatory effects, for example, to induce secretion of IL-10 and IL-1 receptor antagonist (IL-1ra) [4]. In addition, IL-6 has metabolic regulating functions such as promoting energy use of substrates by degrading fat, and stimulating glucose production in liver [1,2].

During endurance exercise, IL-6 is mainly released from skeletal muscle into the circulation [1,5,6,7]. It has been reported that IL-6 is produced by myocytes during endurance exercise [6,8]. Electrical stimulation of C2C12 cells (a murine-derived skeletal muscle cell line) also stimulates IL-6 production in vitro [9]. However, IL-6 exists in interstitial spaces, as detected by immunohistochemistry [6], suggesting that non-myocyte cells may also produce IL-6. The exercise-induced increase in plasma IL-6 concentration is not attenuated in myocyte-specific IL-6 deficient mice [10]. This finding suggests that either IL-6-producing cells other than myocytes, or IL-6-producing organs other than skeletal muscle may exist. The brain is another source of IL-6 in circulation during exercise [11,12], and adipose tissue releases IL-6 into the circulation after exercise, but not during exercise [13]. Exercise induces liver IL-6 gene expression [14,15,16], but the liver does not release IL-6 into the circulation [17]. Peritendon tissue also produces IL-6 [18]. Although it has been reported that peripheral blood mononuclear cells do not contribute to circulating IL-6 [19,20,21,22], it is unclear which organ contributes most substantially to concentrations of IL-6 in the circulation during exercise.

IL-6 is an important regulator for carbohydrate (CHO) metabolism during exercise. Specifically, it increases glucose uptake in muscle [23] and induces glucose production in the liver [24]. Also, IL-6 production is influenced by energy state such as muscle glycogen content [1] and exogenous CHO supplementation [5,19,25,26,27,28]. CHO supplementation during endurance exercise attenuates the increases of plasma IL-6 concentration [5,19,25,26,27,28] and IL-6 mRNA expression in muscle [25,26]; However, contrasting results have also been reported [5,27]. The effects of CHO supplementation on muscle IL-6 production therefore remain unclear. Febbraio et al. have reported that glucose supplementation during exercise attenuates IL-6 release from muscle but does not inhibit IL-6 mRNA expression in muscle tissue. Arterial plasma IL-6 concentration was also attenuated by only about 50% [5]. There are many questions about the effects of CHO supplementation on muscle IL-6 production.

Monocyte chemotactic protein (MCP)-1 is one of the chemokines involved in recruitment of monocytes [29,30], which differentiate into macrophages at the site of inflammation [31]. Following endurance exercise, MCP-1 increases in the circulation [32,33,34,35,36,37,38], and urine [33,34,35]. In skeletal muscle, MCP-1 gene expression and protein concentration also increase immediately after endurance exercise [36,37], and macrophage infiltrates 24-h after endurance exercise [39,40,41]. Since plasma MCP-1 concentration increases [32,33,34,35,36,37,38] and muscle MCP-1 gene expression or protein concentration [34,35] increase during exercise, macrophages may infiltrate in muscle during exercise. Furthermore, as macrophages have the capacity to produce IL-6 [42], infiltrating macrophages may produce IL-6 in muscle. 

Many previous studies have reported the effects of CHO on muscle IL-6 production during endurance exercise [5,25,26,27]. However, the effects of CHO on IL-6-producing cells in muscle are not measured. To investigate the effects of glucose ingestion on muscle IL-6 production during endurance exercise with a special focus on IL-6-producing cells, especially macrophages, we exercised mice with or without glucose ingestion, and examined changes in IL-6-producing cells in muscle by immunohistochemistry.

## 2. Materials and Methods

### 2.1. Animals

Male C57BL/6J mice (*n* = 30) were purchased from Takasugi Experimental Animals Supply (Kasukabe, Japan) at 8 weeks of age and were housed in the breeding room, with 20:00 to 8:00 set as a dark period and 8:00 to 20:00 set as the photoperiod. All the mice were divided in three groups: Sedentary with water ingestion groups as the control group (Con; *n* = 10), exercise with water ingestion group (Ex; *n* = 10) and exercise with glucose ingestion group (Ex + glucose; *n* = 10). The average body weight of the mice was almost equal in each group (Con: 24.3 ± 0.5 g/mice, Ex: 24.3 ± 0.4 g/mice, Ex + glucose: 24.4 ± 0.4 g/mice, *p* = 0.991 using ANOVA). All of the mice had ad libitum access to standard chow (MF, oriental yeast, Tokyo, Japan) and water. The experimental procedures followed the Guiding Principles for the Care and Use of Animals in the Academic Research Ethical Review Committee of Waseda University and were approved (2018-A098).

### 2.2. Exercise Protocol, Glucose Ingestion and Sampling

One week before the experimental trials, all of the mice (including the Con group) were placed on a motorized treadmill (Natsume, Kyoto, Japan) at 10 m/min and 0% incline for 10 min to familiarize to treadmill running. On the day of experiment, Ex and Ex + glucose groups completed 3 h treadmill running at 24 m/min and 7% incline, and ingested water or 6% glucose solution orally with the dose of 200 µL, 30 min before exercise and hourly during exercise. The Con group as the sedentary group ingested water orally over the same time course as the Ex and Ex + glucose groups. All groups were prohibited to access to food and water on the treadmill during the running experiment.

Mice in the Con group were sacrificed 60 min after the last water ingestion and the Ex group and Ex + glucose groups were sacrificed immediately after exercise under isoflurane inhalational anesthesia (Abbott, Tokyo, Japan). Blood samples were collected in heparin processing vacuum drawing blood pipe (TERUMO, Tokyo, Japan) from the inferior vena cava. Plasma was obtained from blood samples by centrifugation at 1600× *g* for 10 min at 4 °C. In addition, gastrocnemius and soleus muscles were removed, and immediately frozen in liquid nitrogen. All samples were stored at −80 °C until analysis.

### 2.3. Enzyme Linked Immuno Solvent Assay (ELISA) Procedure

Plasma, gastrocnemius and soleus IL-6 concentrations were measured using Mouse IL-6 DuoSet ELISA (#DY406; R&D Systems, Minneapolis, MN, USA) according to the manufacturer’s instructions. To perform ELISA assay, gastrocnemius and soleus muscle tissue was homogenized in tissue protein extraction reagent (T-PER; Pierce, Rocford, IL, USA) containing protease inhibitor (Complete mini protease inhibitor cocktail tablet; Roche Diagnostics, Mannheim, Germany). Then, the homogenate was centrifuged at 10,000× *g* for 15 min at 4 °C and the supernatant was used to measure IL-6 concentration. Gastrocnemius and soleus IL-6 concentrations were related to total protein concentration measured using the Pierce^TM^ BCA Protein Assay Kit (Thermo Fisher Scientific, Rockford, IL, USA) according to the manufacturer’s instructions.

### 2.4. Real-Time Polymerase Chain Reaction (PCR)

Total RNA was extracted from the gastrocnemius and soleus homogenate using the RNeasy Fibrous Tissue Mini Kit (Qiagen, Valencia, CA, USA) according to the manufacturer’s instructions. The purity of the extracted total RNA was measured by the NanoDrop system (NanoDrop Technologies, Wilmington, DE, USA). Total RNA was reverse transcribed to cDNA using the High-Capacity cDNA Reverse Transcription Kit (Applied Biosystems, Foster City, CA, USA) according to the manufacturer’s instructions. PCR was performed using the Fast 7500 real-time PCR system (Applied Biosystems, Foster City, CA, USA) and Fast SYBR Green PCR Master Mix (Applied Biosystems, Foster City, CA, USA). PCR conditions for all genes consisted of one denaturing cycle at 95 °C for 10 min, 40 cycles consisting of denaturing at 95 °C for 3 s, and annealing and elongation at 60 °C for 15 min. β-actin RNA was used as the housekeeping gene. All the data were normalized to the housekeeping gene using the ΔΔCt method. All data were expressed as fold change relative to the values of the Con group. The primer sequences used in this study were as follows. *β-actin*; 5’-GCGGACTGTTACTGAGCTGCGT-3’ (Forward) and 5’-TGCTGTCGCCTTCACCGTTCC-3’ (Reverse), *IL-6*; 5’-AACGATGATGCACTTGCAGA-3’ (Forward) and 5’-TGGTACTCCAGAAGACCAGAGG -3’ (Reverse), *Mcp-1*; 5’-CTTCTGGGCCTGCTGTTCA-3’ (Forward) and 5’-CCAGCCTACTCATTGGGATCA-3’ (Reverse).

### 2.5. Immunohistochemistory

Frozen tissues of 6 μm thickness were obtained from gastrocnemius. Frozen tissues were obtained by soaking samples into isopentane which precooled at −150 °C. Serial sections were fixed in 4% paraformaldehyde for 7 min and blocking by 1% bovine serum albumin (BSA) solution for 30 min at room temperature. Anti-F4/80 (ab6640; Abcam, Cambridge, U.K.) and anti-IL-6 (AF406; R&D Systems, Minneapolis, MN, USA) primary antibodies diluted in 1% BSA solution were incubated with the sections overnight at 4 °C. Alexa Fluor 488 donkey anti-rat IgG (A-21208; Thermo Fisher Scientific, Rockford, IL, USA) and Alexa Fluor 555 donkey anti-goat IgG (ab150130; Abcam, Cambridge, U.K.) antibodies diluted in 1% BSA solution were incubated with the sections for 1 h at room temperature. The concentration of the antibodies was 15 μg/mL for IL-6 and 10 μg/mL for F4/80, Alexa Fluor 488 and Alexa Fluor 555. 

The stained sections of the muscle tissue were visualized by fluorescence microscopy (KEYENCE, Osaka, Japan). F4/80-positive and F4/80 and IL-6 double-positive cells were counted in three random 200× magnification fields per slide to derive the average value for each section. F4/80-positive cells and F4/80 and IL-6 double-positive cells were detected with visual judgment of the observer.

### 2.6. Statistical Analysis

Data are presented as mean ± standard error (SE). Data were analyzed using one-way analysis of variance (ANOVA). When ANOVA indicated significant difference, Tukey’s post-hoc test was performed to determine the significance among the means. Statistical significance was defined as *p* < 0.05. Statistical analysis was done using SPSS V25.0 (IBM Japan, Ltd, Tokyo, Japan).

## 3. Results

### 3.1. Glucose Ingestion Inhibits Increase of Plasma IL-6 Concentration Induced by Endurance Exercise

**Figure 1:**
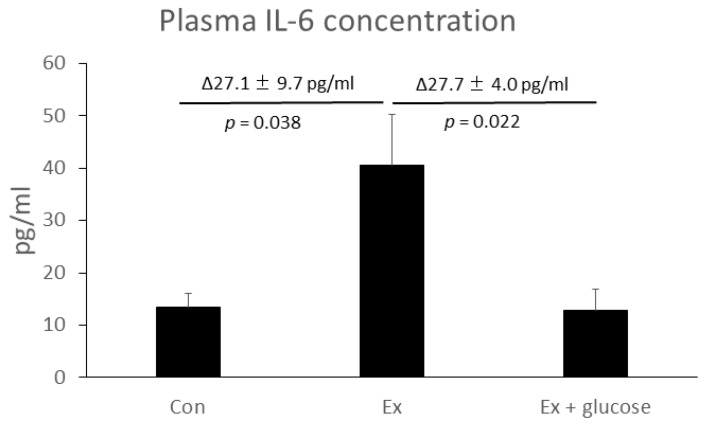
Effects of exercise and glucose ingestion on plasma Interleukin (IL)-6 concentration. The data are presented as means ± standard error (SE). The differences in IL-6 concentration between Ex and Con or Ex + glucose are presented Δ means ± SE above the bar. *p*-values for the differences between Ex and Con or Ex + glucose are presented below the bar.

Firstly, we performed ELISA to evaluate plasma IL-6 concentration. As shown in Figure 1, plasma IL-6 concentration increased in Ex compared to Con (Con: 13.3 ± 2.8 pg/mL vs. Ex: 40.5 ± 9.7 pg/mL, Δ27.1 ± 9.7 pg/mL, *p* = 0.038). Glucose ingestion attenuated the increase of plasma IL-6 concentration (Ex: 40.5 ± 9.7 pg/mL vs. Ex + glucose: 12.8 ± 4.0 pg/mL, Δ27.7 ± 4.0 pg/mL, *p* = 0.022).

### 3.2. Effects of Exercise and Gucose Ingestion on IL-6 mRNA Expression in Gastrocnemius and Soleus

**Figure 2:**
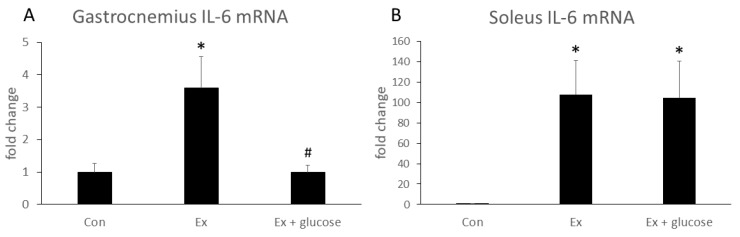
Effects of exercise and glucose ingestion on IL-6 mRNA expression in (**A**) gastrocnemius and (**B**) soleus. The data are presented as the fold change ± SE, relative to the values from the Con. * *p* < 0.05 compared to Con. # *p* < 0.05, compared to Ex.

We performed PCR analysis to investigate the effects of glucose ingestion on the relative difference in IL-6 gene expression from Con as baseline in different types of muscle. As shown in Figure 2, IL-6 mRNA expression of gastrocnemius increased in Ex compared to Con (Con: 1.0 ± 0.3-fold vs. Ex: 3.6 ± 1.0-fold, *p* = 0.019). Glucose ingestion attenuated the increase of IL-6 mRNA expression (Ex: 3.6 ± 1.0-fold vs. Ex + glucose: 1.0 ± 0.2-fold, *p* = 0.015). In soleus, although IL-6 mRNA expression increased in Ex compared to Con (Con: 1.0 ± 0.1-fold vs. Ex: 107.8 ± 33.5-fold, *p* = 0.043), glucose ingestion did not attenuate the increase of soleus IL-6 mRNA expression (Con: 1.0 ± 0.1-fold vs. Ex + glucose: 104.7 ± 35.6-fold, *p* = 0.050; Ex: 107.8 ± 33.5-fold vs. Ex + glucose: 104.7 ± 35.6-fold, *p* = 0.997).

### 3.3. Effects of Exercise and Glucose Ingestion on IL-6 Protein Concentraion in Gastrocnemius and Soleus

**Figure 3:**
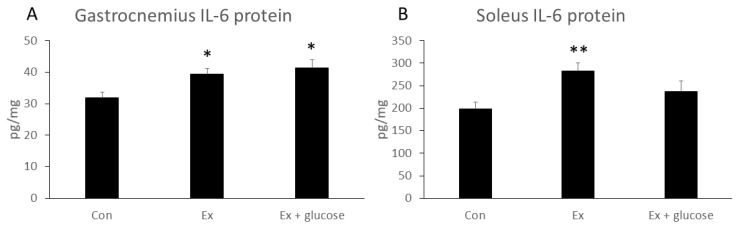
Effects of exercise and glucose ingestion on IL-6 protein concentration in (**A**) gastrocnemius and (**B**) soleus. The data are presented as means ± SE. * *p* < 0.05, ** *p* < 0.01 compared to Con.

We next performed ELISA assay to evaluate IL-6 protein concentration in gastrocnemius and soleus. As shown in Figure 3, although gastrocnemius IL-6 protein concentration increased in Ex compared to Con (Con: 31.9 ± 1.6 pg/mg vs. Ex: 39.3 ± 1.7 pg/mg, *p* = 0.040), glucose ingestion did not attenuate the increase in gastrocnemius IL-6 protein concentration (Con: 31.9 ± 1.6 pg/mg vs. Ex + glucose: 41.3 ± 2.5 pg/mg, *p* = 0.010; Ex: 39.3 ± 1.7 pg/mg vs. Ex + glucose: 41.3 ± 2.5 pg/mg, *p* = 0.750). While soleus IL-6 concentration increased in Ex compared to Con (Con: 198.2 ± 15.3 pg/mg vs. Ex: 282.1 ± 18.8 pg/mg, *p* = 0.009), glucose ingestion did not attenuate the increase in soleus IL-6 protein concentration (Con: 198.2 ± 15.3 pg/mg vs. Ex + glucose: 237.2 ± 22.8 pg/mg, *p* = 0.366; Ex: 282.1 ± 18.8 pg/mg vs. Ex + glucose: 237.2 ± 22.8 pg/mg, *p* = 0.253).

### 3.4. Identification of IL-6 Producing Cells in Gastrocnemius

**Figure 4:**
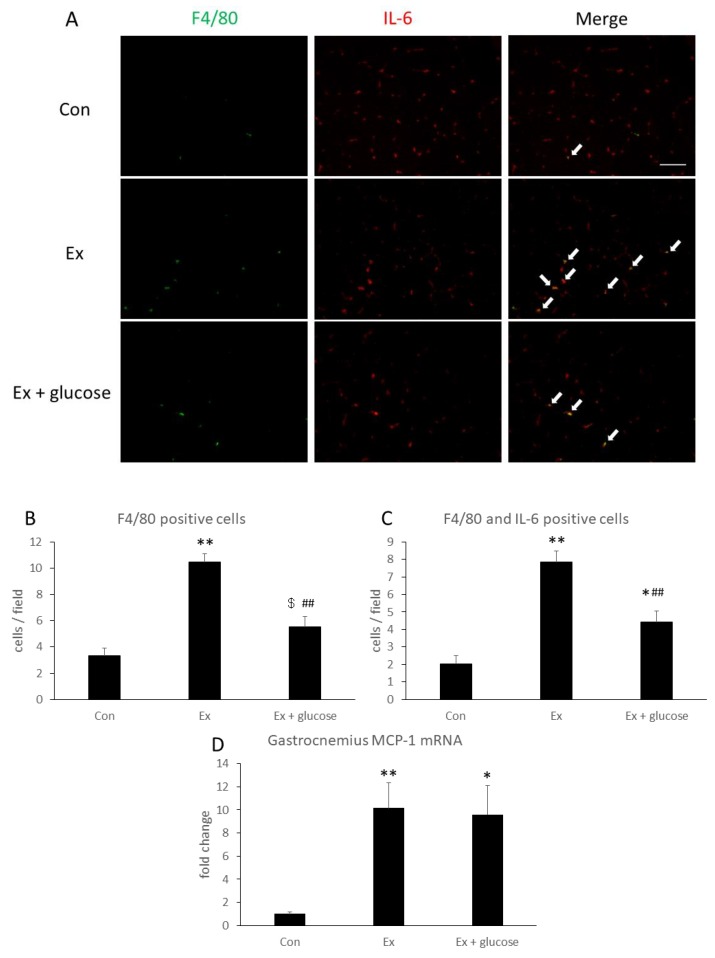
Effects of exercise and glucose ingestion on macrophage infiltration in gastrocnemius. (**A**) Localization of F4/80 [green] and IL-6 [red] of gastrocnemius detected by immunofluorescence staining. Arrows indicate F4/80 and IL-6 double positive cells. Scale bar is 100 µm. (**B**) The number of F4/80 positive cells and (**C**) the number of F4/80 and IL-6 double positive cells. (**D**) Monocyte chemotactic protein (MCP)-1 mRNA expression in gastrocnemius. The data are presented as means ± SE. * *p* < 0.05, ** *p* < 0.01 compared to Con. ## *p* < 0.01, $ *p* = 0.086 compared to Ex.

To identify IL-6 localization and producing cells, we performed immunofluorescence staining in gastrocnemius. As shown in Figure 4A, IL-6 localized with the interstitial space in all groups. Next, we performed double staining of IL-6 and F4/80 as a macrophage marker. As shown in Figure 4A–C, F4/80 positive cells and F4/80- and IL-6-double positive cells increased 3.1- (Con: 3.3 ± 0.6 cells/field vs. Ex: 10.5 ± 0.6 cells/field, *p* < 0.001) or 3.8-fold (Con: 2.0 ± 0.5 cells/field vs. Ex: 7.9 ± 0.6 cells/field, *p* < 0.001) in Ex compared to Con, respectively. By comparison, these cells increased 1.7- (Con: 3.3 ± 0.6 cells/field vs. Ex + glucose: 5.5 ± 0.8 cells/field, *p* = 0.086) or 2.2-fold (Con: 2.0 ± 0.5 cells/field vs. Ex + glucose: 4.4 ± 0.7 cells/field, *p* = 0.033) in Ex + glucose compared to Con, respectively. However, glucose ingestion attenuated the increase of F4/80-positive cells and F4/80- and IL-6-double positive cells by 0.5-fold (Ex: 10.5 ± 0.6 cells/field vs. Ex + glucose: 5.5 ± 0.8 cells/field, *p* < 0.001) or 0.6-fold (Ex: 7.9 ± 0.6 cells/field vs. Ex + glucose: 4.4 ± 0.7 cells/field, *p* = 0.001), respectively. 

Then, we evaluated relative difference in gastrocnemius MCP-1 mRNA expression compared to Con using PCR. As shown in Figure 4D, MCP-1 mRNA expression increased in Ex and Ex + glucose compared to Con, respectively (Con: 1.0 ± 0.2-fold vs. Ex: 10.1 ± 2.2-fold, *p* = 0.007, Con: 1.0 ± 0.2-fold vs. Ex + glucose: 9.6 ± 2.5-fold, *p* = 0.014). 

## 4. Discussion

In the present study, we demonstrated that macrophages infiltrating in muscle during exercise produce IL-6, and glucose ingestion attenuates this response. However, the effects of glucose ingestion on IL-6 production were limited. These results suggest that macrophages may contribute to exercise-induced IL-6 production in muscle during exercise.

It has been reported that when muscle injury is induced by exhaustive exercise or cardiotoxin, MCP-1 is an important factor to induce monocyte/macrophage infiltration in muscle [29,30,40]. In the present study, exercise induced MCP-1 mRNA expression in the gastrocnemius, suggesting that muscle tissue-derived MCP-1 may induce macrophage infiltration in muscle. Although glucose ingestion attenuated macrophage infiltration, glucose ingestion did not attenuate the increase of MCP-1 mRNA expression. One reason for this result is the number of blood monocytes. Our previous study showed that depletion of blood monocytes by clodronate liposome administration inhibited macrophage infiltration after 24 h of exhaustive exercise, even though muscle MCP-1 mRNA expression did not change [41]. This study suggests that not only MCP-1 production, but also the number of blood monocytes is an important factor that regulate macrophage infiltration in muscle. Several studies have reported that the number of blood monocytes increases during endurance exercise, and CHO supplementation attenuates the increase of blood monocytes [19,26,43]. Therefore, attenuation of blood monocytes by glucose ingestion may influence macrophage infiltration in muscle. 

Another reason for why glucose ingestion attenuates macrophage infiltration in muscle is the difference of C-C chemokine receptor type 2 (CCR2) expression by blood monocytes. CCR2 is a major receptor of MCP-1. In addition to MCP-1, CCR2 is a crucial factor for macrophage infiltration in muscle [30,44]. While exercise-induced cortisol is an inducer of CCR2 on monocytes [38], exercise-induced cortisol is attenuated by CHO supplementation [19,25,43]. In the present study, because cortisol-induced CCR2 expression on monocytes may have been inhibited by glucose ingestion, this may also have attenuated macrophage infiltration in muscle. In contrast to our results in which 3 h of endurance exercise induces macrophage infiltration in muscle, Tsuchiya, et al. have reported that walking exercise does not induce macrophage infiltration in muscle [45]. However, it has been reported that vigorous exercise such as 24-h ultraendurance exercise induces macrophage infiltration immediately after exercise [46]. There is a possibility that exercise intensity or time affects macrophage infiltration. 

In the present study, gastrocnemius IL-6 mRNA expression increased significantly during endurance exercise and glucose ingestion attenuated the increase of gastrocnemius IL-6 mRNA expression. However, although soleus IL-6 mRNA expression also increased significantly during endurance exercise, glucose ingestion did not attenuate the increase of soleus IL-6 mRNA expression. Furthermore, gastrocnemius and soleus IL-6 protein concentrations increased during exercise, but glucose ingestion did not attenuate the increase of gastrocnemius and soleus IL-6 protein concentrations. Surprisingly, the correlation between protein and mRNA levels in gastrocnemius was poor (r = 0.195, *p* = 0.329). One reason is that glucose ingestion during exercise may change post-transcriptional regulation of IL-6 in muscle. Febbraio et al. have reported that glucose intake during endurance exercise inhibits IL-6 release from muscle, but IL-6 mRNA expression is not inhibited [5]. In this article, Febbraio et al. proposed that one reason for this result is that glucose intake during endurance exercise may alter post-transcriptional regulation of IL-6 mRNA, such as adjusting translational efficiency or changing IL-6 releasing regulator, although we could not detect it at all [47]. In the present study, in gastrocnemius, IL-6 gene expression increased 3.6-fold and protein concentration increased 1.3-fold during exercise, while in soleus, IL-6 gene expression increased about 100-fold and protein concentration increased only by 1.4-fold. Therefore, in regard to exercise-induced muscle IL-6 production, slow-twitch fiber such as soleus may have low translational efficiency compared to fast-twitch fiber such as gastrocnemius.

In the present study, glucose ingestion attenuated the exercise-induced increase of plasma IL-6 concentration. One reason for this result is that glucose ingestion attenuated IL-6 release from muscle, because it has been reported that the exercise-induced increase of IL-6 in circulation is mainly derived from contracting muscle [7] and glucose intake during exercise inhibits IL-6 release from muscle [5]. However, the mechanism of IL-6 release from muscle is unclear. We have previously reported that an 8-week ketogenic diet inhibited the increase of plasma IL-6 concentration induced by endurance exercise. However, the increase in muscle IL-6 gene expression or protein concentration were not inhibited [48]. Furthermore, several studies reported that the change of IL-6 in muscle and in circulation is not consistent [5,6,10,27]. Furuichi et al. also have reported that IL-6 release from C2C12 cells which is the murine-derived skeletal muscle cell line is induced by electrical stimulation independently of intracellular IL-6 protein levels of C2C12 cells lysate [9]. These studies suggest that muscle IL-6 release is induced independently of the transcription. In the present study, we demonstrated that glucose ingestion attenuates infiltration of IL-6-producing macrophages. Therefore, muscle macrophage-derived IL-6 may contribute to muscle IL-6 release and may be one of the mechanisms for muscle IL-6 release. Another reason for this result is that other organs in addition to muscle may contribute to the increase of IL-6 in circulation. Adipose tissue is one of the candidate as a source of IL-6. Adipose tissue releases IL-6 to the circulation at post 30 min after 1 h endurance exercise, but not immediately after exercise [13]. However, adipose tissue IL-6 gene expression increased immediately after 3 h endurance exercise [49]. This report suggests that during longer duration exercise such as 3 h endurance exercise, adipose tissue may contribute to the increase of IL-6 in circulation. Furthermore, adipose tissue IL-6 gene expression is attenuated by CHO ingestion [49]. In the present study, IL-6 gene expression of epididymal adipose tissue increased by exercise (data not shown), and glucose ingestion attenuated the increase of IL-6 gene expression of epididymal adipose tissue (data not shown). However, measuring only gene expression does not provide any definite information about which organ contributes to IL-6 release into the circulation. 

A limitation of this study is that it could not identify completely what kinds of cells cause change in gene expression or protein production, since homogenate was used for analyses. Although it has been reported that IL-6 gene or protein expression increases in myocytes [6,8,50], it is unclear that whether non-myocyte cells that also exist in muscle tissue (e.g., immune cells, satellite cells, fibroblasts, etc) cause changes in IL-6 gene or protein expression during exercise. In the present study, we demonstrated that infiltrating macrophages produce IL-6 in muscle during endurance exercise. However, we could not reveal the percentage of macrophage-derived IL-6 to total IL-6 production in skeletal muscle tissue. We also observed that glucose ingestion attenuates infiltration of IL-6-producing macrophages, but total muscle IL-6 protein concentration was not attenuated. Therefore, macrophage-derived IL-6 may not contribute substantially to total IL-6 production in skeletal muscle. Because IL-6 was mainly localized in the interstitial space, further study is needed in order to identify IL-6 producing cells, other than macrophages. 

## 5. Conclusions

In conclusion, we demonstrated that macrophages were one type of IL-6-producing cells in muscle during exercise and glucose ingestion inhibited infiltration of IL-6-producing macrophages. However, the effects of glucose ingestion on muscle IL-6 production were limited.
